# Awareness of Epidural Analgesia Among Childbearing Women in Western Region of Saudi Arabia: A Cross-Sectional Study

**DOI:** 10.7759/cureus.42618

**Published:** 2023-07-28

**Authors:** Mohammed A Althubaiti, Azzam A Hanif, Mohammed K Alghamdi, Jawharah Tirkistani, Abdullah M Alobedi, Abdulaziz F Alshareef, Mutaz G Alshaikh, Abdullah F Saeidi, Nawaf A Alazwari, Mokhtar Shatla

**Affiliations:** 1 Medicine and Surgery, Umm Al-Qura University, Makkah, SAU; 2 Family Medicine, Umm Al-Qura University, Makkah, SAU

**Keywords:** epidural anaesthesia, anesthesia, awareness, childbearing woman, western-region of saudi arabia

## Abstract

Background

Labor pain is one of the most excruciating experiences that women can go through. Epidural anesthesia (EDA) is the most prevalent form of labor analgesia considered as a secure and effective method of pain relief for women during active labor. The EDA works by numbing the nerves that cause pain. To our knowledge, only a few studies have been conducted on the use of EDA in Saudi Arabia.

Objectives

The purpose of this study was to assess the awareness of EDA among childbearing women in the western region of Saudi Arabia.

Methods

We carried out a cross-sectional study using a self-administered online questionnaire to measure awareness about EDA. The study targeted the general population of women from the western region of Saudi Arabia who were aged between 18 and 50 years. A total of 1,137 questionnaires were returned and analyzed.

Results

This study assessed the perspectives of 1,137 women. The results revealed that 52.6% of women who received EDA showed a good level of knowledge of the procedure, while 26.1% of women had not experienced EDA (P = 0.001). Good knowledge of EDA was detected among 39.3% of women aged 36-50 years. This rate was significantly higher than that of women younger than 20 years old, of which 24.3% had good knowledge (P = 0.038).

Conclusion

This study shows that women in this particular region have a lack of knowledge about EDA. Therefore, it is recommended that more education about EDA be provided during antenatal visits to improve awareness.

## Introduction

Labor pain is one of the most severe types of pain that women can experience. To deal with labor pain, some women rely on certain pain reduction methods, such as epidural analgesia (EDA) [1], which is a technique that is commonly used in reducing pain during labor [2]. EDA is a technique that involves delivering medication in close proximity to their site of action either at receptors in the spinal cord or at nerves as they leave the spinal cord. The drugs are delivered directly to the targeted area, allowing for stronger pain relief with lower doses of drugs compared to systemic administration. This approach reduces the risk of side effects or toxicity that could occur with systemic administration [3]. In this process, the analgesic agent is injected into the epidural space, which is a specific region of the spine [4]. This procedure induces a complete loss of sensation from the lower abdomen to the feet [3].

Within 20-30 minutes of administering the medication, the pain begins to subside [5]. The length of time in which the pain will be reduced depends on the dosage and type of medication used [6]. EDA produces almost complete labor pain alleviation (90-95%) if given in a timely manner, but once the medication’s duration of action ends, the pain will return [6,7].

International studies conducted in Najera and Hong Kong have determined that awareness of EDA is insufficient among pregnant women [8,9]. The first cross-sectional study conducted in Riyadh in 2014 measured the educational level of primigravid employed women receiving EDA in comparison with unemployed women. The results showed employed pregnant women were a greater tendency to request EDA than non-employed women (46.7% vs. 18.2%) [10]. Furthermore, recent studies conducted in Jeddah and Khamis Mushait have revealed a limited understanding of EDA among pregnant women [11,12]. Surprisingly, there has not been a study conducted in Makkah City with a focus on awareness of EDA. Therefore, the aim of this study was to assess the awareness of EDA among childbearing women in the western region of Saudi Arabia.

## Materials and methods

Study design and participants

This cross-sectional study included the general population of women from the western region of Saudi Arabia (Makkah, Jeddah, Taif, Madinah, Yanbu) who were aged 18 years and above. We excluded women from other regions or those who could not become pregnant due to medical conditions.

Ethical considerations and sample size

Ethical clearance was obtained from the Biomedical Ethics Committee at the College of Medicine, Umm Al-Qura University, Makkah, Saudi Arabia (approval number: HAPO-02-K-012-2023-03-1509). Informed consent was obtained from the study participants after acquainting them with the purpose and nature of the study. A self-administered questionnaire was distributed online to women from the western region of Saudi Arabia between March 2023 and April 2023 via social media platforms (WhatsApp, Twitter, and Telegram) using Google Forms (Google LLC, Mountain View, California, United States). The Raosoft calculator (Raosoft, Inc., Seattle, USA) was used to determine the sample size required for this study with a confidence interval of 95% and a 50% prevalence of the sample size [13]. The minimum sample size was calculated to be 385, and we recruited 1,137 participants.

Study tool

A web-based questionnaire was developed based on the reviewed literature [12]. The electronic survey consisted of four sections, which included questions about the demographic data of the participants (e.g., age, gender, nationality, and education level), history of exposure to EDA, and frequency of epidural usage. The survey also included questions related to knowledge and future education preferences. A structured self-administered questionnaire was designed in English and translated into Arabic and was reviewed by a panel of experts to resolve any discrepancies. A pilot study was conducted to test the feasibility and applicability of the questionnaire. Twenty women were surveyed before starting the data collection and modifications were made, their responses were excluded from the analysis of the main data. Knowledge of the EDA questionnaire was developed and validated by the investigators based on previous research [12].

Statistical analysis

Data were collected, reviewed, and entered into the Statistical Package for Social Sciences version 21 (IBM© SPSS© Statistics version 21, IBM© Corp., Armonk, NY, USA). All statistical methods used were two-tailed with an alpha level of 0.05. A P-value less than or equal to 0.05 was considered significant. The overall knowledge levels regarding EDA were categorized as poor if the respondent’s score was less than 60%, and a good level of knowledge was indicated by an overall score of 60% or higher.

Descriptive analysis was performed by calculating the frequency distribution and percentages for the study variables, including the women’s personal data, obstetric histories, frequencies of epidural usage, histories of exposure, and future education preferences. In addition, knowledge regarding EDA was tabulated, and overall knowledge regarding EDA was graphed. Cross-tabulation of factors associated with the women’s knowledge of EDA was carried out with the Pearson chi-squared test for significance and the exact probability test if there were small frequency distributions.

## Results

A total of 1,137 childbearing women fulfilled the inclusion criteria and completed the study survey. The participants’ ages ranged from 18 to 50 years with a mean age of 31.5 ± 12.9 years. The vast majority of the study women were Saudi (85.8%; 975). Regarding educational level, 883 (77.7%) were university graduates, and 254 (22.3%) had a secondary level of education or less. A total of 568 women (50%) were not working, 322 (28.3%) were non-healthcare workers, and 247 (21.7%) were healthcare workers. Of these, 494 (43.4%) had previously had a normal vaginal delivery, and 391 (34.3%) had previously had a cesarean section (Table 1).

**Table 1:** Socio-demographic data of study participants in the western region of Saudi Arabia

Demographic data	No	%
Age in years		
< 20	74	6.5%
20-35	613	53.9%
36-50	450	39.6%
Nationality		
Saudi	975	85.8%
Non-Saudi	162	14.2%
Educational level		
Secondary/below	254	22.3%
University graduate	883	77.7%
Work		
Not working	568	50.0%
Non-health care field	322	28.3%
Health care field	247	21.7%
History of normal vaginal delivery		
Never	643	56.6%
1/more	494	43.4%
History of cesarean section		
Never	746	65.6%
1/more	391	34.4%

The women were asked to reveal how often they supposed that EDA was used during labor in Saudi Arabia, and less than a third (32.8%) believed that EDA was used often by women, and 27.3% believed that it was occasionally used. A total of 19.8% believed that it was commonly used, and only 8.6% believed it was rarely used to control labor pain. However, the rest of the women (11.5%) were unsure about the rate of EDA use. Additionally, the women were asked whether they had a previous encounter with EDA and how they would rate their experience with pain control using this method. There were 439 women (38.6%) who had experienced EDA at least once during their previous deliveries, and the rest of them (61.4%) reported that they had never experienced it (Table 2).

**Table 2:** Frequency of epidural usage and history of exposure among study women in the western region of Saudi Arabia

Epidural anesthesia	No	%
How common do you think epidural analgesia is used during labor?		
Rarely	98	8.6%
Occasionally	310	27.3%
Often	373	32.8%
Commonly	225	19.8%
Not sure	131	11.5%
Have you ever had epidural anesthesia?		
Yes	439	38.6%
No	698	61.4%

The women were also asked to respond to questions that assessed their awareness and knowledge of four main points regarding EDA with “true,” “false,” or “do not know.” The topics of these questions included whether contractions become weaker with EDA, whether the insertion process of the epidural needle is very painful, whether EDA is convenient and eases delivery by allowing women to push when needed, and whether EDA causes paraplegia as a complication. A chi-squared test of uniform distribution was used to analyze the women’s responses and to determine whether they differed from the expected equal distribution of responses for each answer (goodness of fit, “true” = 128 women, “false” = 128 women, and “do not know” = 128 women).

A total of 45.4% responded that they did not know whether contractions became weak or stopped completely after the administration of EDA. Fewer agreed with this claim (42.7%), and 11.9% disagreed with it (P = 0.001). Also, the women’s responses differed from expected when asked whether the epidural insertion process was more painful than the labor pain itself. Almost half the respondents (52%) disagreed, 36.3% did not know, and only 11.7% agreed (P = 0.001). Furthermore, 46.8% thought that the epidural reduced labor pain and allowed the mother to push when needed, while 38.4% did not know, and only 14.8% disagreed with this claim (P = 0.001). Lastly, 42.6% were uncertain about whether the epidural causes paraplegia, and 44.1% believed it was not true, while only 13.4% believed that EDA truly can cause paralysis (Table 3).

**Table 3:** Women's knowledge about epidural anesthesia in the western region of Saudi Arabia EPA: epidural analgesia

Knowledge items	True		False		I don't know	
	No	%	No	%	No	%
Contractions become weak or stop completely after administration of EPA	486	42.7%	135	11.9%	516	45.4%
Epidural insertion is more painful than the labor pain itself	133	11.7%	591	52.0%	413	36.3%
Epidural reduce labor pain and allow the mother to push when needed	532	46.8%	168	14.8%	437	38.4%
Epidural causes paraplegia	152	13.4%	501	44.1%	484	42.6%

**Figure 1:**
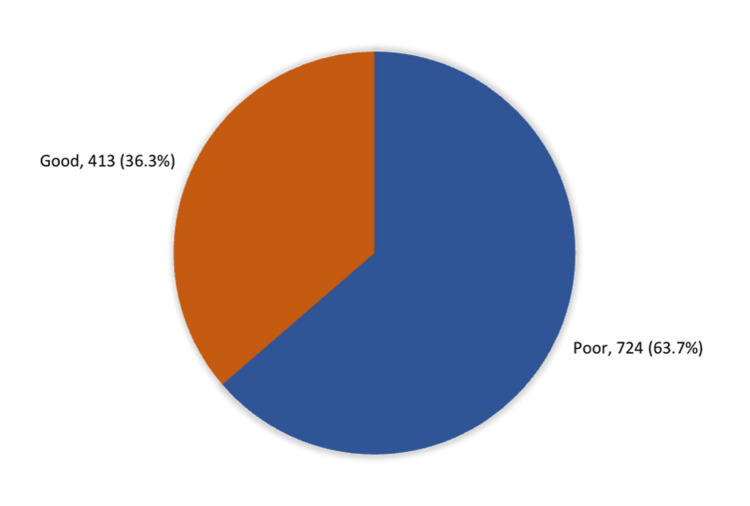
Overall women's knowledge about epidural anesthesia in the western region of Saudi Arabia

There were 413 (36.3%) women who had an overall good level of knowledge regarding EDA, while 724 (63.7%) had an overall poor knowledge level (Figure 1).

Good knowledge of EDA was detected among 39.3% of women aged 36-50 years but only 24.3% of women younger than 20 years, and the difference between these two rates was statistically significant (P = 0.038). In addition, 39.5% of university graduates had an overall good knowledge level, whereas only 25.2% of women with a lower educational level had a similar level of knowledge (P = 0.001).

Good knowledge was detected among 49% of the women working in the healthcare field versus 30.8% of housewives (P = 0.001). Exactly 40.5% of the women with a history of normal vaginal delivery and 43.7% of those with a history of cesarean section showed good knowledge of EDA (P = 0.001). Furthermore, 52.6% of those who had EDA showed a good knowledge level of the procedure, in comparison to 26.1% of women who did not receive EDA (P = 0.001) (Table 4).

**Table 4:** Factors associated with women's knowledge of epidural anesthesia

Factors	Overall knowledge level				p-value
	Poor		Good		
	No	%	No	%	
Age in years					0.038*^$^
< 20	56	75.7%	18	24.3%	
20-35	395	64.4%	218	35.6%	
36-50	273	60.7%	177	39.3%	
Nationality					0.082
Saudi	611	62.7%	364	37.3%	
Non-Saudi	113	69.8%	49	30.2%	
Educational level					0.001*
Secondary/below	190	74.8%	64	25.2%	
University graduate	534	60.5%	349	39.5%	
Work					0.001*
Not working	393	69.2%	175	30.8%	
Non-health care field	205	63.7%	117	36.3%	
Health care field	126	51.0%	121	49.0%	
History of normal vaginal delivery					0.011*
Never	430	66.9%	213	33.1%	
1/more	294	59.5%	200	40.5%	
History of cesarean section					0.001*
Never	504	67.6%	242	32.4%	
1/more	220	56.3%	171	43.7%	
Have you ever had epidural anesthesia?					0.001*
Yes	208	47.4%	231	52.6%	
No	516	73.9%	182	26.1%	

The women were asked if they would prefer to have an educational session before undertaking EDA, and most indicated that they would like to attend such a session during antenatal classes (81.7%), while the others (18.3%) did not think it was needed. Those who liked the idea indicated a preference for receiving such education through physician consultation sessions (40.8%), special sessions offered by the anesthetist (39.1%), video (8.8%), and written pamphlets (5%) (Table 5).

**Table 5:** Future education preference of epidural anesthesia in the western region of Saudi Arabia

	No	%
If you are considering the epidural analgesia in labor, do you prefer it to be introduced formally during your antenatal visits?		
Yes	929	81.7%
No	208	18.3%
If you would like future education/introduction on epidural anesthesia, how?		
During doctor’s consultation	464	40.8%
In a special session by the anesthetist	445	39.1%
By means of readable pamphlets	57	5.0%
By means of video that you can watch	100	8.8%
Does not require future introduction	71	6.2%

## Discussion

This study examined individuals who lived in the western region of Saudi Arabia to assess their knowledge, awareness, and practices regarding EDA for labor. Prior studies have revealed variations in public awareness and understanding of EDA [8,14-16]. Surprisingly, our results showed that most women of childbearing age in the western region of Saudi Arabia have a low awareness level of EDA. Only 36.3% of the women had an overall good knowledge level regarding EDA, while 63.7% had an overall poor knowledge level. This result is consistent with the findings of other studies that were recently conducted in Saudi Arabia. There is a study done in Riyadh city and Khamis Mushait which reported 87.4% and 62.5% respectively of the participants had poor knowledge [11,17].

Compared to previous studies in Saudi Arabia, pregnant or women who had experienced labor provided the most replies in the study sample. A previous study conducted in Riyadh and Khobar, Saudi Arabia, reported that women were highly knowledgeable about EDA [14-15]. In contrast, a study conducted in Abu Dhabi found that women were not well educated about EDA [18].

In Babil, Iraq, and Karachi, Pakistan, it was found that women had limited overall knowledge about the role of EDA in labor [19,20]. Moreover, a study conducted in Nigeria found that only 19.5% of Nigerian women knew about EDA [16]. Similarly, in Hong Kong, Williams found a lack of knowledge among pregnant women about EDA in labor [8]. The discrepancy in the level of knowledge and acceptance of EDA in labor possibly be accounted for by the fact that in undeveloped nations, delivery is considered as a physiological process requiring slight interference. Additionally, a lack of antenatal follow-up and education during antenatal visits may be responsible for the low level of awareness [12].

Consequently, several misconceptions are associated with the use of EDA for labor pain. According to our study, the most commonly believed misconception was that “contractions become weak or stop completely after the administration of EDA,” with 42.7% of respondents believing this to be true. Similarly, the most common misconception was that EDA could cause paraplegia in Jeddah, Khamis Mushait, and Riyadh, but not in Khobar [11,12,15,17]. Paralysis remains one of the main concerns: 42.6% of the women in our study were not sure whether EDA causes paraplegia, and 44.1% believed this was not true, while 13.4% believed EDA truly could cause paralysis.

Furthermore, 46.8% of the women thought that EDA reduces labor pain and allows the mother to push when needed, which is considered a high rate when compared with the corresponding rates of 40.6% in Jeddah and 30% in Jazan, the variation in the percentage between the cities could be due to increasing use of EDA nowadays which lead to rising of the awareness among the parous women in comparison with the past. However, the rate is lower than the rate of 59.8% reported in Riyadh [12,14,21]. When asked if the epidural insertion was more unpleasant than the actual labor discomfort, the responses from women varied. Only 11.7% of the patients agreed with this statement, while 52.3% of patients disagreed and 36.3% were unsure.

This study showed that 38.6% of women had an EDA at least once during their previous labor. This rate is higher than that reported in a previous study conducted in the eastern region of Saudi Arabia, which indicated that only 22.6% of women had an EDA [22]. The level of awareness could be associated with a variety of factors, including age, educational level, and prior exposure to EDA. We found that 52.6% of women who received EDA showed a good level of knowledge of the procedure, whereas 26.1% of women who did not experience EDA had a good level. Both Mohammed et al. and Alahmari et al. reported similar findings [11,20].

We also found that women between 36 and 50 years of age had a higher awareness of EDA than the other age groups. This is consistent with a study conducted in Nigeria, which reported that awareness was more common among older women [19]. It was also found that a higher educational level was associated with more knowledge about and acceptance of EDA. These results are similar to previous studies conducted in Khamis Mushait and Jazan in Saudi Arabia and in Nigeria [11,16,21]. However, they contrast with findings in India, where it has been reported that educational level did not positively correlate with EDA awareness [23].

The level of knowledge has a significant impact on the decision to either accept or reject EDA in current and future pregnancies. Accordingly, to raise people’s awareness of EDA and reduce their concerns about the procedure, the women were asked if they would have preferred an educational session before receiving EDA. The vast majority (81.7%) of the participants agreed with the need for an educational session during antenatal classes. In addition, 40.8% of the women who liked the idea reported a preference for EDA being presented and explained to them by a physician during their antenatal visits, in a special session by the anesthetist (39.1%), video (8.8%), and readable pamphlets (5%).

Thus, it is recommended that prenatal educational sessions be established in regard to possible pain management during labor, including EDA. Additionally, healthcare practitioners should put more effort into social media and provide the community with evidence-based information about possible methods of pain relief during labor since social media currently plays a key role in community transformation.

Limitations

This study has a few limitations. First, the study was limited to the western region of Saudi Arabia. Therefore, it cannot be generalized to other areas. Second, the cross-sectional design precludes the ability to make causal conclusions.

## Conclusions

This study demonstrated a lack of knowledge and comprehension regarding EDA among women in western Saudi Arabia, especially those who were younger and had a low educational level. To increase the level of awareness among populations regarding EDA, we encourage the use of multiple media platforms to enhance awareness through periodic educational campaigns. We also recommend that all expectant women receive routine education at antenatal visits about the use of EDA during vaginal delivery, which could be provided by either obstetricians or anesthetists.
